# Interrupted Time Series Analysis of Alcohol Use Disorder Treatment Utilisation During the Coronavirus Pandemic in Hamburg, Germany

**DOI:** 10.1111/dar.70107

**Published:** 2026-01-27

**Authors:** Jakob Manthey, Carolin Kilian, Ludwig Kraus, Anna Schranz, Bernd Schulte

**Affiliations:** ^1^ Centre for Interdisciplinary Addiction Research, Department of Psychiatry and Psychotherapy University Medical Center Hamburg‐Eppendorf Hamburg Germany; ^2^ Department of Psychiatry, Medical Faculty University of Leipzig Leipzig Germany; ^3^ National Institute of Public Health University of Southern Denmark Copenhagen Denmark; ^4^ Danish Institute for Advanced Studies University of Southern Denmark Odense Denmark; ^5^ Department of Public Health Sciences, Centre for Social Research on Alcohol and Drugs Stockholm University Stockholm Sweden; ^6^ Institute of Psychology ELTE Eötvös Loránd University Budapest Hungary

**Keywords:** addiction, disruption, healthcare, intervention, medication, withdrawal

## Abstract

**Introduction:**

Alcohol use and treatment for alcohol use disorder (AUD) changed during the pandemic. Alcohol consumption increased among high‐risk groups, while treatment utilisation declined, raising concerns about an expanding treatment gap. This study analyses inpatient and outpatient AUD treatment trends during the pandemic.

**Methods:**

We analysed electronic health records from 5671 patients residing in Hamburg, Germany, who utilised at least one alcohol‐related treatment between January 2016 and December 2021. Data from two statutory health insurance providers and two pension funds provided weekly numbers of patients receiving AUD treatment, including outpatient (psychiatric consultations, psychotherapy, pharmacotherapy), inpatient standard and intensive treatment, and outpatient and inpatient rehabilitation programs. Using a segmented regression approach, we examined changes over time for: (i) any treatment; (ii) outpatient treatment; and (iii) inpatient treatment across five periods of the pandemic: pre‐pandemic, first lockdown, between lockdowns, second lockdown and post‐second lockdown.

**Results:**

During the first lockdown in spring 2020, AUD treatment utilisation declined by 27% before briefly returning to near pre‐pandemic levels. A gradual decline was observed in later pandemic periods, even after containment measures were lifted. The decline was more pronounced in inpatient settings than in outpatient settings, at −45% and −15%, respectively, during the first lockdown.

**Discussion and Conclusions:**

The utilisation of AUD treatment services dropped substantially during the pandemic, particularly in inpatient settings. This reduction may have contributed to the rise in alcohol‐related deaths during this period. This study is the first to assess long‐term inpatient and outpatient treatment trends across multiple pandemic phases.

## Key Points

- Interrupted time series analyses assessed COVID‐19's impact on alcohol use disorder treatment.
- In spring 2020, alcohol use disorder treatment utilisation dropped abruptly by 27%.
- Immediate declines were greater for inpatient (45%) than outpatient (15%) treatment.
- Gradual declines persisted in 2021 after containment measures were eased.

## 1 Introduction

The COVID‐19 pandemic and the public health measures to contain the viral spread have had a considerable and long‐term impact on alcohol use and attributable harm. In many high‐income countries, alcohol‐specific mortality has increased considerably [1, 2]. To explain this detrimental trend, two major explanations have been brought forward: changes in alcohol use patterns and changes in treatment utilisation.

Changes in alcohol use patterns have been described comprehensively. A meta‐analysis found that many individuals had fewer occasions to drink early in the pandemic, resulting in an average decline of self‐reported alcohol use [3, 4]. Contrasting this overall population trend, individuals with heavy drinking patterns or at risk for alcohol use disorders (AUDs) were more likely to increase their alcohol use during the pandemic [5, 6]. With this polarisation of alcohol use patterns during the pandemic, sales of alcoholic beverages have remained largely stable (see, e.g., data from 14 European countries: [1]).

Changes in AUD treatment utilisation during the pandemic have been assessed using survey and administrative data. In the United States of America, survey data showed a decline in self‐reported treatment utilisation in 2020, followed by a rebound to previous levels in 2022 [7]. Following the declaration of a national emergency in March 2020, psychotherapy for AUD declined by approximately 50%, while pharmacotherapy remained unaffected, according to administrative US data [8]. In Europe, data from Germany suggest that inpatient AUD treatment declined in March 2020 and the following months of that year [9]. In Andalusia (Spain), a decline in outpatient treatment utilisation for addiction was observed during the first lockdown (March to June 2020; [10]). Examining a small German sample of AUD patients treated in outpatient settings, a drop in treatment utilisation was observed between April 2020 and July 2021 [11], while no change in alcohol‐related contacts was seen in an inpatient psychiatric treatment facility in Sweden [12].

In summary, most research has pointed to a decline in AUD treatment utilisation during the COVID‐19 pandemic, particularly in the early periods. This is concerning, as it implies a widening of the already large treatment gap for AUD [13]. However, previous studies are limited by short observation periods and a focus on specific treatment types (e.g., only outpatient or inpatient treatment). We aimed to close this gap by analysing the trajectories of AUD treatment utilisation for a broad set of interventions delivered in inpatient and outpatient settings up to December 2021 in the city‐state of Hamburg, Germany. We hypothesised that AUD treatment utilisation in inpatient settings was more affected by the COVID‐19 pandemic and respective countermeasures, as services in outpatient settings were easier to maintain with physical distancing measures in place.

## 2 Methods

### 2.1 Data Sources

Electronic health records from two major statutory health insurance providers (SHI; Allgemeine Ortskrankenkasse [AOK] Rheinland/Hamburg—Die Gesundheitskasse, Deutsche Angestelltenkrankenkasse [DAK—Gesundheit]) and two German pension insurance providers (Deutsche Rentenversicherung Nord; Deutsche Rentenversicherung Bund) were linked for the years 2016 to 2021. The two SHI cover about 25% of the adult population in Hamburg. SHI data comprise records of addiction‐related outpatient and inpatient treatments and outpatient prescription data. The pension insurance data include information on addiction‐specific outpatient and inpatient rehabilitation services. Due to the absence of a universal identifier across the four sources, a linking procedure was implemented to generate cryptographically protected human identifiers derived from demographic information (first name, last name, year of birth, gender), thereby enabling linkage of the respective datasets into a unified analytical set. Details have been described elsewhere [14].

### 2.2 Study Population and Interventions Considered

Among adults aged 18–99 years covered by one of the two SHIs at any time during the period 2016 to 2021, we included all individuals utilising at least one alcohol‐related intervention between 1 January 2016 and 31 December 2021. As outlined in Table 1, we considered a broad set of alcohol‐related interventions conducted in both outpatient and inpatient settings. Comprehensive information on treatment definitions is published elsewhere [15].

**TABLE 1:** Description of seven treatment types analysed.

Source	Setting	Description of care
SHI	Outpatient	Brief psychiatric or psychological consultation (majority of contacts are with psychiatrists)
SHI	Outpatient	Short‐ or long‐term psychotherapy
SHI	Outpatient	Prescription of alcohol‐specific medications (Acamprosate, Naltrexone, Nalmefene)
SHI and PF	Outpatient	Rehabilitation treatment in outpatient settings (including ‘Full‐day outpatient care’)
SHI and PF	Inpatient	Rehabilitation treatment in inpatient settings
SHI	Inpatient	Standard inpatient treatment: mostly somatic care; no qualified withdrawal treatment
SHI	Inpatient	Intensive inpatient treatment (qualified withdrawal treatment): somatic and psychosocial care

Abbreviations: PF, pension funds; SHI, statutory health insurance.

### 2.3 Primary and Secondary Outcomes

The primary outcome was a weekly aggregation of the number of patients in any treatment in that week. As secondary outcomes, we aggregated the number of patients in: (i) outpatient treatment (brief psychiatric consultation, short‐term and long‐term psychotherapy, pharmacotherapy, rehabilitation treatment, see Table 1); and (ii) inpatient treatment (standard or intensive inpatient treatment, rehabilitation treatment). Quarterly and monthly aggregations were examined descriptively (see Figures S1 and S2).

We aimed to include any treatment starting or ending during the study period. However, long‐term rehabilitation treatment episodes funded by the pension funds ending outside the study period, that is, after 2021, were not available for analyses. Thus, treatment utilisation was underestimated towards the end of the time series, in particular, the second half of 2021. To address this bias, sensitivity analyses on primary and secondary outcomes without rehabilitation treatments were conducted.

### 2.4 Pandemic Periods

For the analyses, we aimed to define periods that reflected both infection dynamics and measures to contain the spread of the virus. In Germany, including the city‐state of Hamburg, there were five infection waves: spring 2020, winter 2020/2021, spring 2021, fall 2021 and winter 2021/2022 [16]. The containment measures in Germany—and any other country—were documented by the Oxford COVID‐19 Government Response Tracker (OxCGRT) between 2020 and 2022 [17]. The OxCGRT stringency index captures eight indicators for containment and closure (school closing, workplace closing, cancellation of public events, restrictions on gathering size, close public transport, stay‐at‐home requirements, restrictions on internal movement, restrictions on international travel) as well as public information campaigns and ranges from 0 (no restrictions) to 100 (maximum restrictions). During the five infection waves, increased values of the OxCGRT stringency index were documented (see Figures S3 and S4). Based on the overlap of infection rates and containment measures, a threshold of 70 in the OxCGRT stringency index was chosen to define five periods, which were largely concordant with definitions in previous studies [18, 19]: (i) pre‐pandemic; (ii) first wave/restriction in spring 2020 (henceforth: ‘lockdown 1’); (iii) relaxation in summer 2020; (iv) restrictions during second and third wave (henceforth: ‘lockdown 2’); and (v) fourth wave with less restrictions (see Table S1, for details). For simplicity, we use the term ‘lockdown’ in this text, but we acknowledge that the containment measures implemented in Germany were relatively modest compared to those in other countries. They did not entail strict curfews or significant restrictions on movement.

### 2.5 Statistical Analyses

To determine changes in treatment utilisation across the five periods, a segmented regression approach was used for an interrupted time series (ITS) analysis. Upon a first examination of the primary outcome across the five periods (see Figures S1 and S2), the following model was developed:

(1) $$\begin{array}{c} Y_{t}=\beta_{0}+\beta_{1}\cdot \mathrm{Tim}e_{t}+\beta_{2}\cdot \text{Lockdown}1_{t}\\ \;+\beta_{3}\cdot \text{TimeSinceLockdown}1_{t}\\ \;+\beta_{4}\cdot \text{TimeSinceBetweenLockdown}s_{t}\\ \;+\beta_{5}\cdot \text{Lockdown}2_{t}+\beta_{6}\cdot \text{TimeSinceLockdown}2_{t}\\ \;+\beta_{7}\cdot \text{TimeSinceAfterLockdown}s_{t}+\varepsilon_{t} \end{array}$$

where $\beta_{1}$ denotes the underlying time trend (continuous variable: number of weeks from 1 to 314); $\beta_{2}$ denotes the immediate (level) change during Lockdown 1 (spring 2020; dummy variable: 1 during this period, 0 otherwise); $\beta_{3}$ denotes the gradual (slope) change from the start of lockdown 1 onward (continuous variable: 0 before lockdown 1, then counts 1, 2, 3,… for each subsequent week, including after the period ends); $\beta_{4}$ denotes the gradual (slope) change from the start of the between‐lockdowns period onward (continuous variable: 0 before between‐lockdowns, then counts 1, 2, 3,… for each subsequent week); $\beta_{5}$ denotes the immediate (level) change during lockdown 2 (winter 2020/2021; dummy variable: 1 during this period, 0 otherwise); $\beta_{6}$ denotes the gradual (slope) change from the start of lockdown 2 onward (continuous variable: 0 before lockdown 2, then counts 1, 2, 3,… for each subsequent week); $\beta_{7}$ denotes the gradual (slope) change from the start of the after‐lockdowns period (Summer 2021) onward (continuous variable: 0 before after‐lockdowns, then counts 1, 2, 3,… for each subsequent week) and $\varepsilon_{t}$ is the error term.

This formula reflects a ‘time since’ approach, which allows modelling of the sustained impact of each period on subsequent periods, under the assumption that the effects of one period may persist and that periods are not completely independent from each other. This is an extension of the classic segmented regression approach (see, e.g., [20]). In our model, only the two lockdown periods are represented by dummy variables ($\beta_{2}$ and $\beta_{5}$), as the remaining periods did not show evidence of immediate level changes. Gradual (slope) changes over time are modelled for each period ($\beta_{1},\beta_{3},\beta_{4},\beta_{6},\beta_{7}$), with each ‘time since’ variable continuing to accumulate after its respective period begins.

For the primary and each secondary outcome, model fitting involved two or three steps: first, a baseline model with GLM was fitted according to Equation (1). Second, a generalised additive regression model (GAM) was fitted to account for annual seasonality via cubic splines (*k* = 20 knots). Residuals of each GAM model were inspected using visual means to identify seasonality, stationarity and autocorrelation. Third, generalised additive mixed models (GAMM) were fitted to account for autocorrelation—if present. ACF and pACF plots as well as the *auto.arima* function of the *forecast* package version 8.24.0 [21] were used to determine autoregressive and moving average terms to be included in GAMM. For the final models (GAM or GAMM), ACF and pACF plots are shown in Figures S5 and S6.

To determine the percentage reduction for lockdown periods with significant changes, we compared the observed treatment utilisation against a counterfactual scenario without a lockdown [−(1−(observed/counterfactual))]. This counterfactual was estimated using the ITS model with a value 0 in the corresponding covariates (for lockdown 1: $\text{Lockdown}1_{t}$ and $\text{TimeSinceLockdown}1_{t}$).

All analyses were conducted in R version 4.5.0 [22]. The data cannot be shared, but the R codes are available publicly (https://github.com/jakobmanthey/PRAGMA_covid).

## 3 Results

### 3.1 Description of Sample

We included *n* = 5671 patients seeking alcohol‐related treatment between 2016 and 2021 (for annual estimates, see Table S2). The sample contained *n* = 1796 (31.7%) women (*n* = 3875; 68.3% men). In 2020, the sample was on average 52.5 years old (median: 53 years; interquartile range (IQR): 43 to 61 years).

Over the 6‐year period, *n* = 3096 patients (54.6%) received at least one brief outpatient psychiatric consultation for AUD, *n* = 112 patients (2.0%) completed at least one short‐ or long‐term outpatient psychotherapy, and *n* = 272 patients (4.8%) were prescribed at least one outpatient AUD medication. A total of *n* = 1790 patients (31.6%) entered standard and *n* = 1921 (33.9%) entered intensive inpatient AUD treatment. Finally, *n* = 525 patients (9.3%) participated in at least one outpatient, and *n* = 564 (9.9%) in at least one inpatient rehabilitation program for AUD.

Between 2016 and 2019, between *n* = 2159 (2018) and *n* = 2246 (2017) patients were registered in AUD treatment utilisation.

### 3.2 Primary Outcome: Overall Treatment Utilisation

**FIGURE 1:**
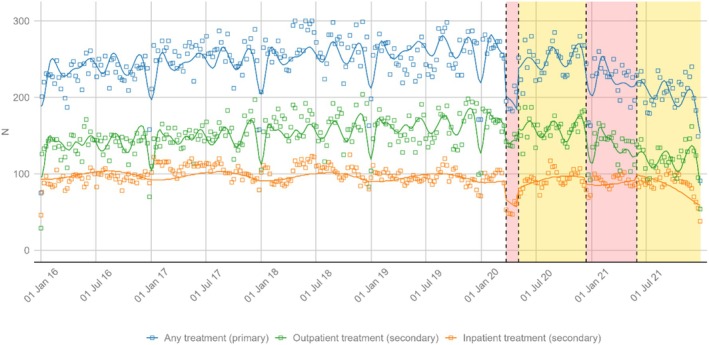
Weekly number of patients in any alcohol‐related treatment and by inpatient/outpatient setting. Plotted are observed (rectangles) and predicted (lines) values from regression models. Shaded areas: Red = lockdown periods (23 March 2020 to 4 May 2020; 14 December 2020 to 31 May 2021), yellow = periods with fewer restrictions (4 May 2020 to 14 December 2020; 31 May 2021 to 31 December 2021).

On average, *n* = 243 (median: 247; IQR: 227 to 264) patients were utilising AUD treatment per week between 2016 and 2021. According to the ITS, there was an immediate (−52.4) and gradual (−5.2 per week) decrease in the number of patients in AUD treatment during lockdown 1 (22 March to 3 May 2020; see Figure 1). In a counterfactual scenario without lockdown 1, the model estimates a weekly median of 267 patients (IQR 263 to 269) in AUD treatment during this period. Compared to the observed values, this corresponds to a 27.3% (IQR −30.2 to −23.5%) reduction in AUD treatment utilisation. After the first lockdown, the weekly number of patients in AUD treatment gradually increased again (+5.8 per week), balancing out the negative trend during lockdown 1. During lockdown 2, a gradual decrease (−2.6 per week) in the number of patients in AUD treatment was registered. No other statistically significant changes were observed in the primary outcome (see model summary in Table 2).

**TABLE 2:** Summary of regression models for main analyses.

Predictors	Primary outcome: Any AUD treatment^a^			Secondary outcome: Outpatient AUD treatment^b^			Secondary outcome: Inpatient AUD treatment^c^		
	Estimates	95% CI	*p*	Estimates	95% CI	*p*	Estimates	95% CI	*p*
Intercept	**233.96**	**226.46–241.45**	**< 0.001**	**136.06**	**131.57–140.55**	**< 0.001**	**99.63**	**90.39–108.87**	**< 0.001**
Time	**0.14**	**0.09–0.20**	**< 0.001**	**0.15**	**0.11–0.18**	**< 0.001**	−0.02	−0.09 to 0.05	0.534
Lockdown1	**−52.42**	**−77.89** to **−26.94**	**< 0.001**	**−19.34**	**−35.67** to **−3.01**	**0.020**	**−23.32**	**−34.62** to **−12.02**	**< 0.001**
TimeSinceLockdown1	**−5.24**	**−8.93** to **−1.55**	**0.006**	−1.67	−3.88 to 0.55	0.139	−2.40	−6.13 to 1.33	0.207
TimeSinceBetweenLockdowns	**5.78**	**1.19–10.38**	**0.014**	1.41	−1.35 to 4.17	0.316	3.03	−1.48 to 7.54	0.187
Lockdown2	−2.62	−21.44 to 16.20	0.784	4.21	−7.50 to 15.91	0.480	−7.53	−18.07 to 3.00	0.160
TimeSinceLockdown2	**−2.62**	**−4.54** to **−0.70**	**0.008**	**−1.41**	**−2.56** to **−0.25**	**0.017**	−0.69	−2.61 to 1.22	0.478
TimeSinceAfterLockdowns	1.36	−0.64 to 3.35	0.181	**1.42**	**0.22** to **2.62**	**0.021**	−0.99	−3.08 to 1.09	0.350
Smooth term (week)			**< 0.001**			**< 0.001**			**0.020**
Observations (weeks)	314			314			314		
*R* ^2^	0.542			0.579			0.341		

*Note:* Statistically significant effects are highlighted in bold font.

Abbreviations: AUD, alcohol use disorder; CI, confidence interval.

^a^ Results of a generalised additive mixed model (GAMM) controlling for autoregression with a MA(1) term.

^b^ Results of a generalised additive model (GAM).

^c^ Results of a generalised additive mixed model (GAMM) controlling for autoregression with an AR(1) and MA(2) term.

The overall decline in AUD treatment utilisation during the pandemic can also be seen in annual figures. Compared to 2019, 5% and 11% fewer people utilised any AUD treatment in 2020 and 2021, respectively (see Table S2).

### 3.3 Secondary Outcomes: Outpatient and Inpatient Treatment Utilisation

On average, *n* = 148 (median 148; IQR 136 to 164) and *n* = 96 (median 96; IQR 89 to 104) patients were seeking outpatient and inpatient AUD treatment per week between 2016 and 2021, respectively. Before the first lockdown, there was a statistically significant increase in AUD treatment utilisation in outpatient (+0.15 per week) but not in inpatient settings.

During the first lockdown, ITS findings suggest immediate reductions in both outpatient (−19.3) and inpatient (−23.3) treatment utilisation, and no statistically significant gradual reductions (see Table 2). Using the counterfactual of no lockdown as reference, the relative reductions were greater for inpatient (−45.2%; IQR −48.3% to −37.3%) as compared to outpatient settings (−14.6%; IQR −16.4% to −13.1%). For outpatient settings only, there was a gradual decline during the second lockdown (−1.4 per week), which was nullified in the period after the second lockdown (+1.4 per week).

### 3.4 Sensitivity Analyses

In sensitivity analyses, we examined AUD treatment utilisation without rehabilitation treatment, resulting in a smaller weekly number of patients in any (median: *n* = 176), outpatient (median: *n* = 111) and inpatient (median: *n* = 65) treatment across the time series (see also Figure S7). We found statistically significant immediate reductions of AUD treatment utilisation with similar effect sizes as in the main analyses in any (−33.0%; IQR −36.3% to −26.9%), outpatient (−19.5%; IQR: −21.7% to −18.2%) and inpatient (−55.9%; IQR: −57.7% to −42.8%) settings during lockdown 1 (see model summary in Table S3). While there were gradual reductions in outpatient settings during lockdown 2 according to the main analyses, the sensitivity analyses suggest immediate reductions of AUD treatment utilisation during lockdown 2 in inpatient settings (−9.6; compared to counterfactual: −11.4%; IQR: −23.7 to −8.3%). As rehabilitation treatment was excluded in the sensitivity analyses, the findings imply immediate declines in standard or intense (withdrawal) therapy.

## 4 Discussion

### 4.1 Summary

In Hamburg, Germany, AUD treatment utilisation declined abruptly during the first lockdown in spring 2020. After the first lockdown, the number of patients in AUD treatment recovered to nearly pre‐lockdown levels; however, a decline in AUD treatment utilisation was observed during later periods of the pandemic. Overall, AUD treatment utilisation appeared to be lower towards the end of 2021 as compared to 2019.

As hypothesised, stronger declines in AUD treatment utilisation were observed in inpatient compared to outpatient settings. This pattern is broadly consistent with available studies on this subject evaluating AUD treatment utilisation in either outpatient or inpatient settings [7, 8, 9, 10, 11, 12]. Our study, however, is the first to examine a broad range of AUD treatment services up until the end of 2021.

### 4.2 Limitations

This retrospective study makes use of electronic health records from about 5600 patients seeking treatment for AUD. The data were not collected for the purpose of this study, but for reimbursement of health services. Biases, such as misclassification and deliberate omission of diagnoses for alcohol use problems, cannot be excluded and can have an impact on the number of patients estimated to utilise AUD treatment. However, if these biases do not vary systematically over time, for example, AUD diagnoses are more likely to be misclassified or omitted during stressful periods, such as lockdowns, our main findings would not be affected.

### 4.3 Implications

Our findings clearly demonstrate that AUD treatment utilisation was adversely and sustainably affected by the COVID‐19 pandemic. Before we discuss the consequences of this trend, we want to offer potential explanations for the decline.

According to an international review of the literature, only about one in six people with AUD enter formal treatment [13]. A range of barriers have been identified for treatment utilisation at the patient, provider and system levels [23], including but not limited to stigmatisation, knowledge and problem‐recognition [24]. It can be assumed that many of the well‐established barriers have not changed during the pandemic and cannot explain our findings. It appears likely that the decline in both outpatient and inpatient AUD treatment utilisation is a combination of patient and system‐level factors. On the one hand, patients may have wanted to avoid places with increased infection risks during the first lockdown, such as public transport, but also hospitals and psychiatric practices. On the other hand, service provision was reported to be affected in different ways. In Ireland, a reduced number of patients in group therapy was reported [25], while a German ad hoc survey suggested that detoxification treatment was stopped altogether in 12% of clinics [26]. With regards to rehabilitation programs, the German pension funds recommended not admitting any patients for 10 days into rehabilitation programs at the beginning of the first lockdown, yet outpatient services could be continued via telehealth [27]. US data also suggests only brief disruptions in outpatient AUD treatment utilisation [28].

The available evidence on disruptions of treatment services is mostly confined to the first lockdown. Possibly, health professionals and institutions were better prepared for subsequent infection waves and confinement measures. This would also explain why, during the second lockdown, we observed less pronounced immediate changes in AUD treatment utilisation. Yet, this would not explain why we see gradual and persistent reductions of AUD treatment utilisation in 2021, even after lockdown periods. It is plausible to assume that in 2021, most services have been adapted to the new situation.

However, on the patient side, persistently low treatment utilisation may have continued even after containment measures were eased. Throughout 2021, rates of infection and cases of the virus remained very high (see Figure S4). During this time, the fear of infection may have remained elevated, reducing patients' willingness to access public services, including transport and healthcare. Moreover, fear of infection may have also reduced social contact with family, friends and colleagues, which allows alcohol‐related problems to remain undetected for longer.

Consistent with our hypothesis, the decline of AUD treatment utilisation was greater for inpatient as compared to outpatient settings. We argue that this could be explained by containment measures having more severe restrictions on inpatient settings as compared to outpatient settings. While inpatient treatment does require personal contact, some outpatient interventions (e.g., psychiatric consultation or psychotherapy; prescription) could be continued using telehealth. However, in California, a transition to telehealth among people with AUD could be observed primarily for those who have not previously been in treatment [29]. Possibly, patients with AUD requiring more intense interventions that could not be conducted through telehealth or those without access to digital means, for example, people experiencing homelessness, may have benefited less from these new treatment forms.

Overall, those who utilise AUD treatment are likely to have a longer history of heavy drinking, a greater AUD severity and suffer from mental health comorbidities [30]. In other words, the treatment utilising population comprises those who require it the most, and temporary suspension of treatment may have detrimental, long‐term consequences. Integrated care models that involve a close, seamless provision of a range of treatment options are considered to be best practice [23], but could not be maintained during some periods of the pandemic, according to our findings. Suboptimal AUD treatment access can pose elevated long‐term health risks (e.g., cancer mortality [31]). Thus, it is not surprising that the year 2020 constituted a turning point in annual alcohol‐specific mortality rates in Germany and many other countries [1, 2]. Between 2016 and 2019, the age‐standardised alcohol‐specific mortality rate declined gradually from 18.4 to 16.5 per 100,000 population, but they increased to 17.3, 17.4 and 18.3 between 2020 and 2022 [32]. In 2023 and 2024, these rates have returned to pre‐pandemic levels of 17.8 and 17.2 per 100,000 population, respectively.

Our findings should also be viewed in the context of studies investigating treatment access for and mortality from other conditions. In the United States of America, the number of older people accessing cancer care, including screening and therapy, has declined markedly during the COVID‐19 pandemic [33]. Using data from England, UK, a decline in cancer access was linked to a subsequent increase in cancer mortality [34]. Also in England, hospitalisations for heart failure declined in March 2020 and were followed by increases in out‐of‐hospital heart failure deaths [35]. A pattern of declining hospitalisations and a subsequent increase in mortality for various chronic conditions other than COVID‐19 was also observed in Denmark [36]. These studies support our hypothesis that the decline in AUD treatment utilisation during the pandemic may have negative consequences for public health, including increased mortality.

## 5 Conclusion

During the COVID‐19 pandemic, utilisation of AUD treatment dropped substantially, and this was more pronounced in inpatient settings (e.g., detoxification treatment). Suboptimal treatment access may be one explanation for increased alcohol‐specific mortality during the pandemic.

## Author Contributions

**Jakob Manthey:** conceptualisation, methodology, software, formal analysis, resources/data curation, writing – original draft, visualisation, funding acquisition. **Anna Schranz:** resources/data curation. **Carolin Kilian:** software. **Bernd Schulte:** supervision/project administration, funding acquisition. All authors: validation, investigation, writing – review and editing.

## Funding

This publication is based on the project ‘Patient Routes of People with Alcohol Use Disorder in Germany’ (PRAGMA) which was funded by the Innovation Committee of the Federal Joint Committee (Gemeinsamer Bundesausschuss, GBA) under the funding code 01VSF21029. The funder was not involved in the study design, collection, analysis, interpretation of data or preparation of this publication.

## Conflicts of Interest

Jakob Manthey reports financial support provided by AOK and World Health Organization. He also reports a relationship with Deutsche Hauptstelle für Suchtfragen that includes speaking and lecture fees and a relationship with German Parliament that includes paid expert testimony. The other authors declare no conflicts of interest.

## Supporting information


**Figure S1:** Trend of people in AUD treatment per quarter.
**Figure S2:** Trend of people in AUD treatment per month.
**Figure S3:** Stringency index of the Oxford COVID‐19 Government Response Tracker per day.
**Figure S4:** Trend of COVID‐19 cases and deaths per week.
**Figure S5:** Results of autocorrelation function (ACF) and partial autocorrelation function (pACF) of residuals from main analyses.
**Figure S6:** Results of autocorrelation function (ACF) and partial autocorrelation function (pACF) of residuals from sensitivity analyses.
**Figure S7:** Weekly number of patients in any alcohol‐related treatment and by inpatient/outpatient setting (sensitivity analyses). Plotted are observed (rectangles) and predicted (lines) values from regression models. Shaded areas: red = lockdown periods (23 March 2020 to 4 May 2020; 14 December 2020 to 31 May 2021), yellow = periods with fewer restrictions (4 May 2020 to 14 December 2020; 31 May 2021 to 31 December 2021).
**Table S1:** Definitions of time periods.
**Table S2:** Annual number of people utilising AUD treatment.
**Table S3:** Model summary of sensitivity analyses.

## Data Availability

The underlying data and R codes are publicly available (https://github.com/jakobmanthey/PRAGMA_covid).
