# Supplementary material for: Interrupted Time Series Analysis of Alcohol Use Disorder Treatment Utilisation During the Coronavirus Pandemic in Hamburg, Germany
Source: Drug Alcohol Rev. 2026 Jan 27;45(1):e70107. doi: 10.1111/dar.70107 (PMC12839788; doi:10.1111/dar.70107)
Supplement: Supplementary file 1 — Figure S1: Trend of people in AUD treatment per quarter. Figure S2: Trend of people in AUD treatment per month. Figure S3: Stringency index of the Oxford COVID‐19 Government Response Tracker per day. Figure S4: Trend of COVID‐19 cases and deaths per week. Figure S5: Results of autocorrelation function (ACF) and partial autocorrelation function (pACF) of residuals from main analyses. Figure S6: Results of autocorrelation function (ACF) and partial autocorrelation function (pACF) of residuals from sensitivity analyses. Figure S7: Weekly number of patients in any alcohol‐related treatment and by inpatient/outpatient setting (sensitivity analyses). Plotted are observed (rectangles) and predicted (lines) values from regression models. Shaded areas: red = lockdown periods (23 March 2020 to 4 May 2020; 14 December 2020 to 31 May 2021), yellow = periods with fewer restrictions (4 May 2020 to 14 December 2020; 31 May 2021 to 31 December 2021). Table S1: Definitions of time periods. Table S2: Annual number of people utilising AUD treatment. Table S3: Model summary of sensitivity analyses. [file DAR-45-0-s001.docx]

**Title:** Utilization of alcohol use disorder treatment during the COVID-19 pandemic in Hamburg, Germany

**Authors:** Jakob Manthey, Carolin Kilian, Ludwig Kraus, Anna Schranz, Bernd Schulte

Supplementary Figure 1. Trend of people in AUD treatment per quarter


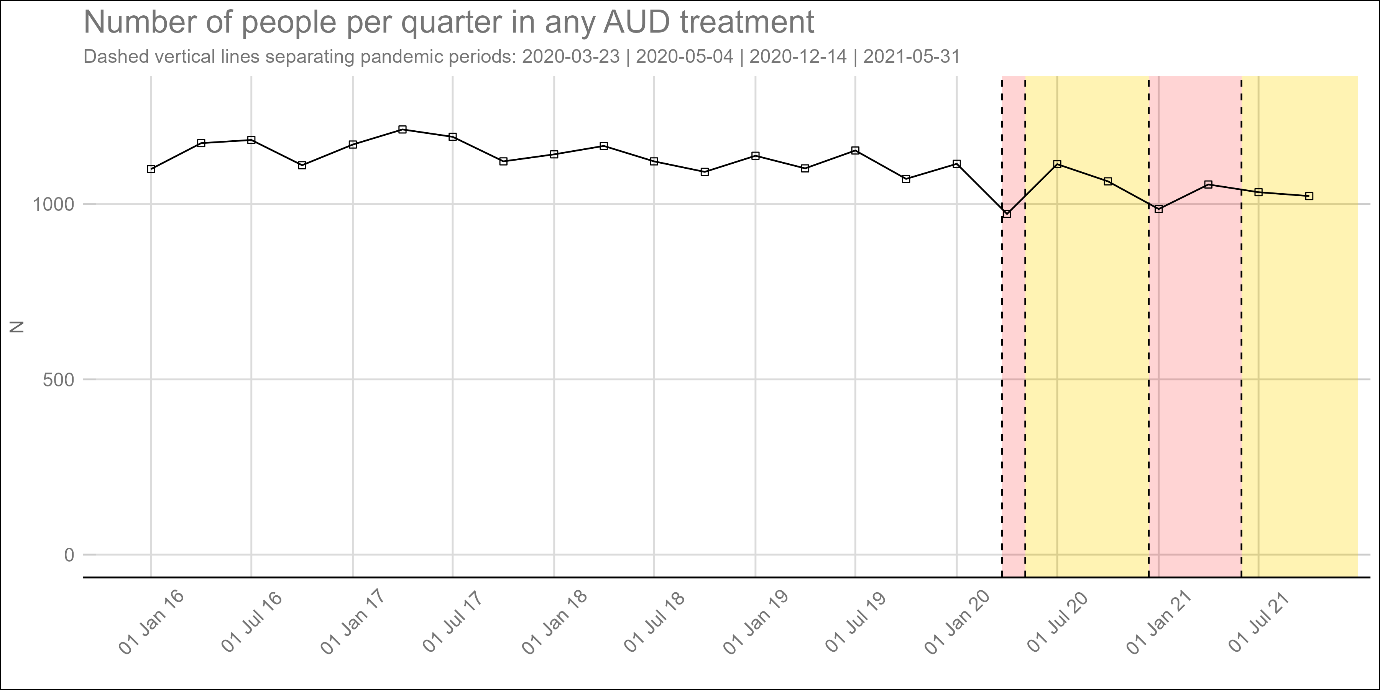


Supplementary Figure 2. Trend of people in AUD treatment per month


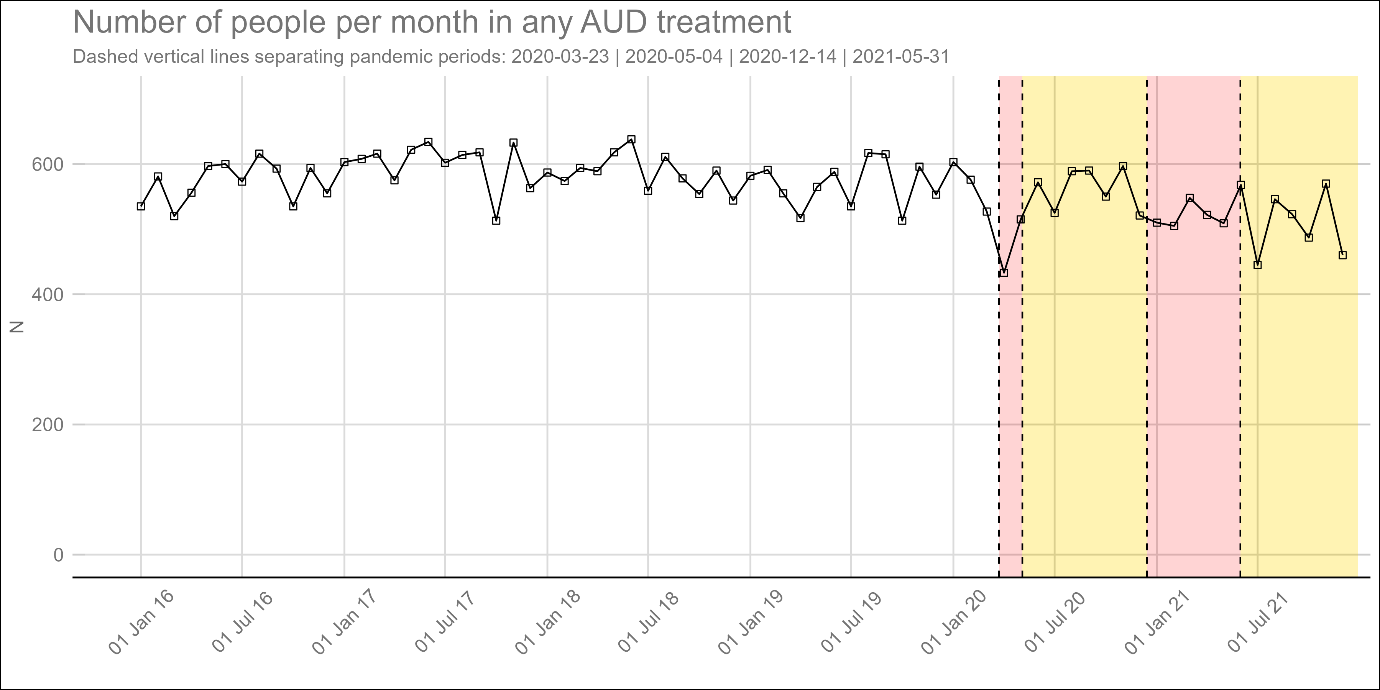


Supplementary Figure 3. Stringency index of the Oxford COVID-19 Government Response Tracker per day


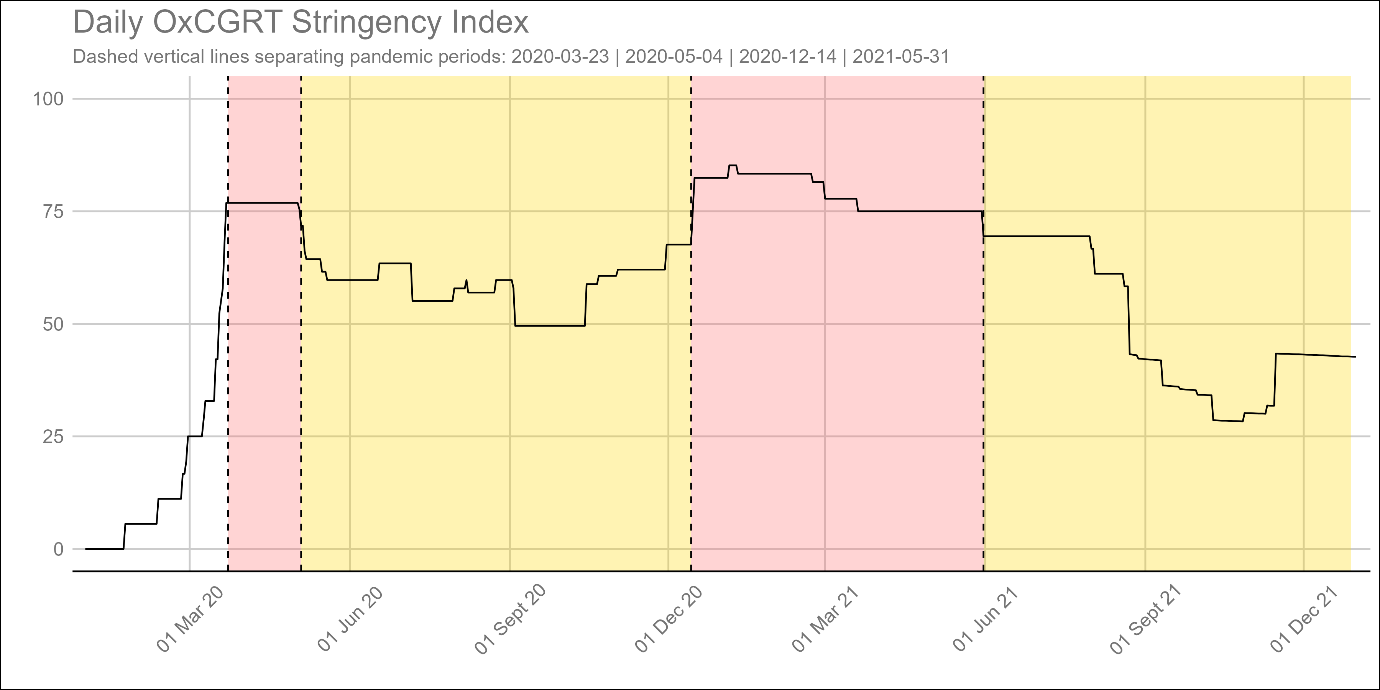


Supplementary Figure 4. Trend of COVID-19 cases and deaths per week


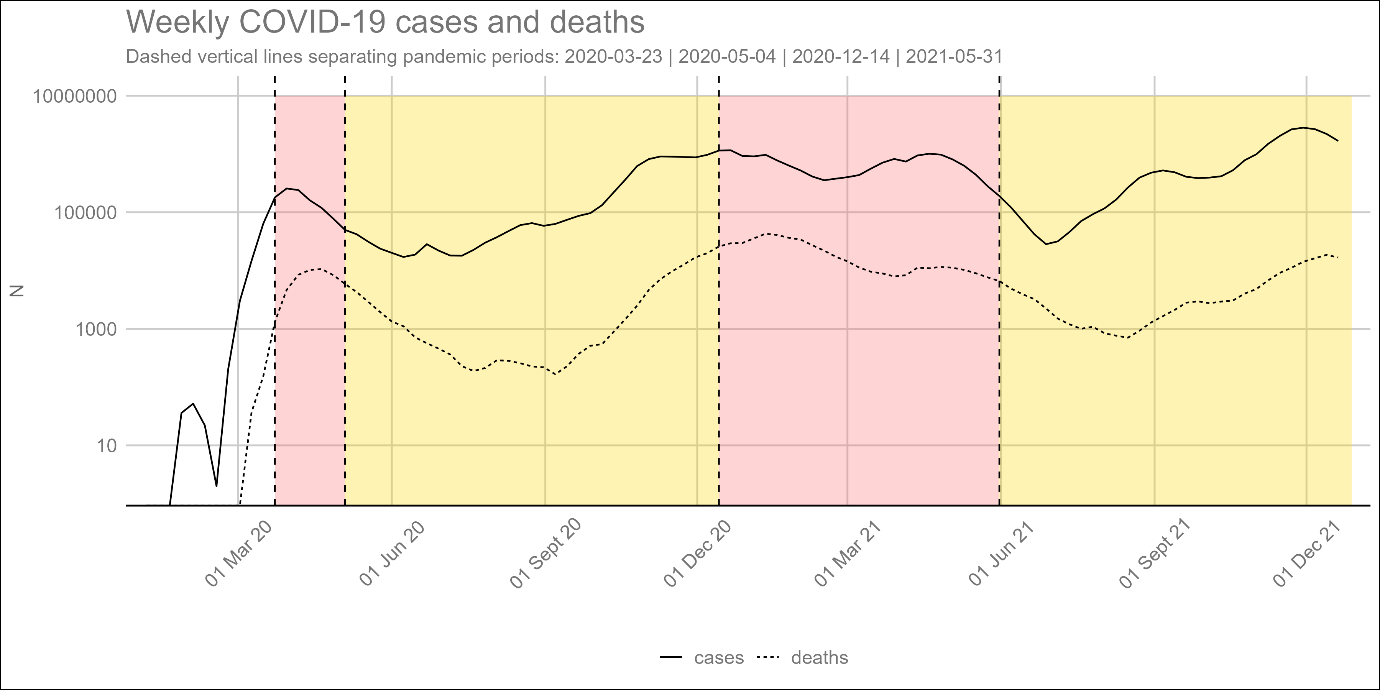


Supplementary Figure 5. Results of autocorrelation function (ACF) and partial autocorrelation function (pACF) of residuals from main analyses


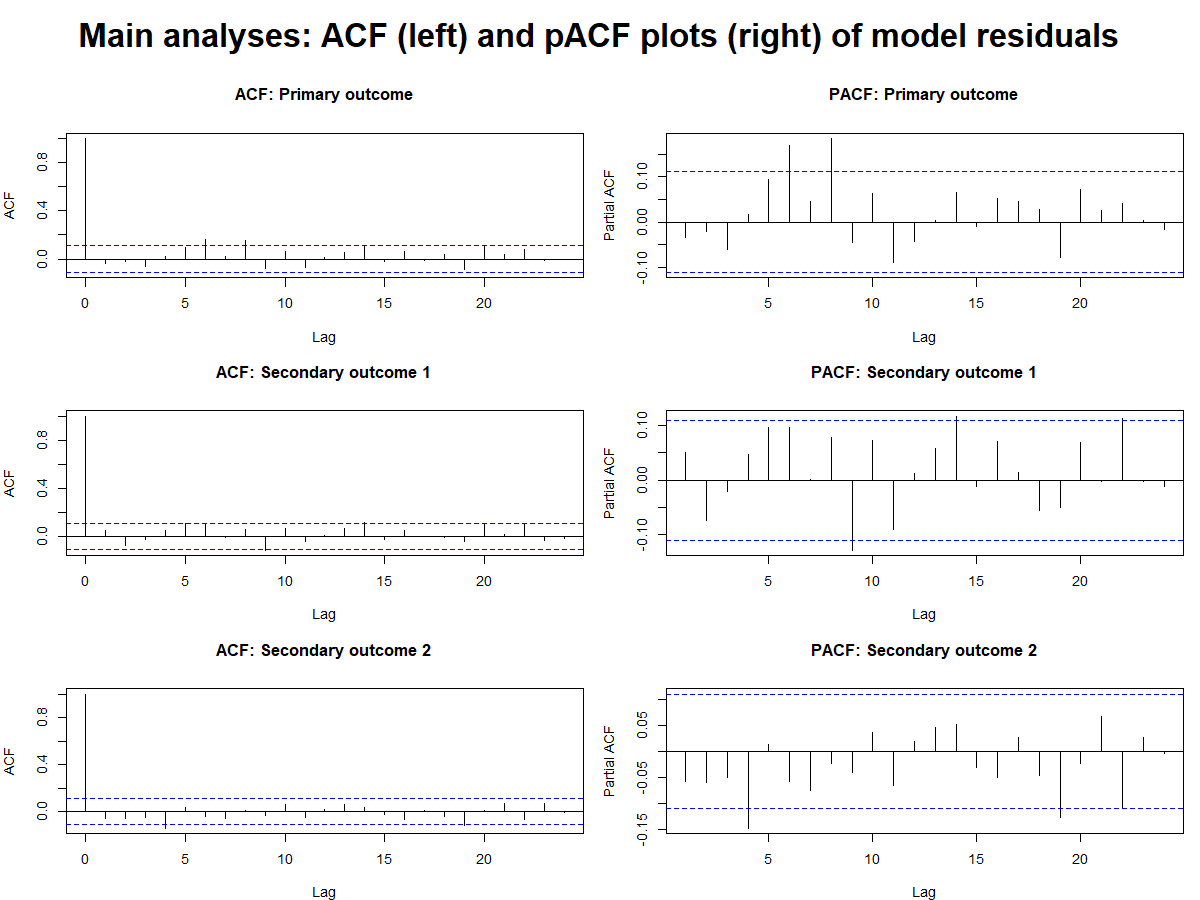


Supplementary Figure 6. Results of autocorrelation function (ACF) and partial autocorrelation function (pACF) of residuals from sensitivity analyses


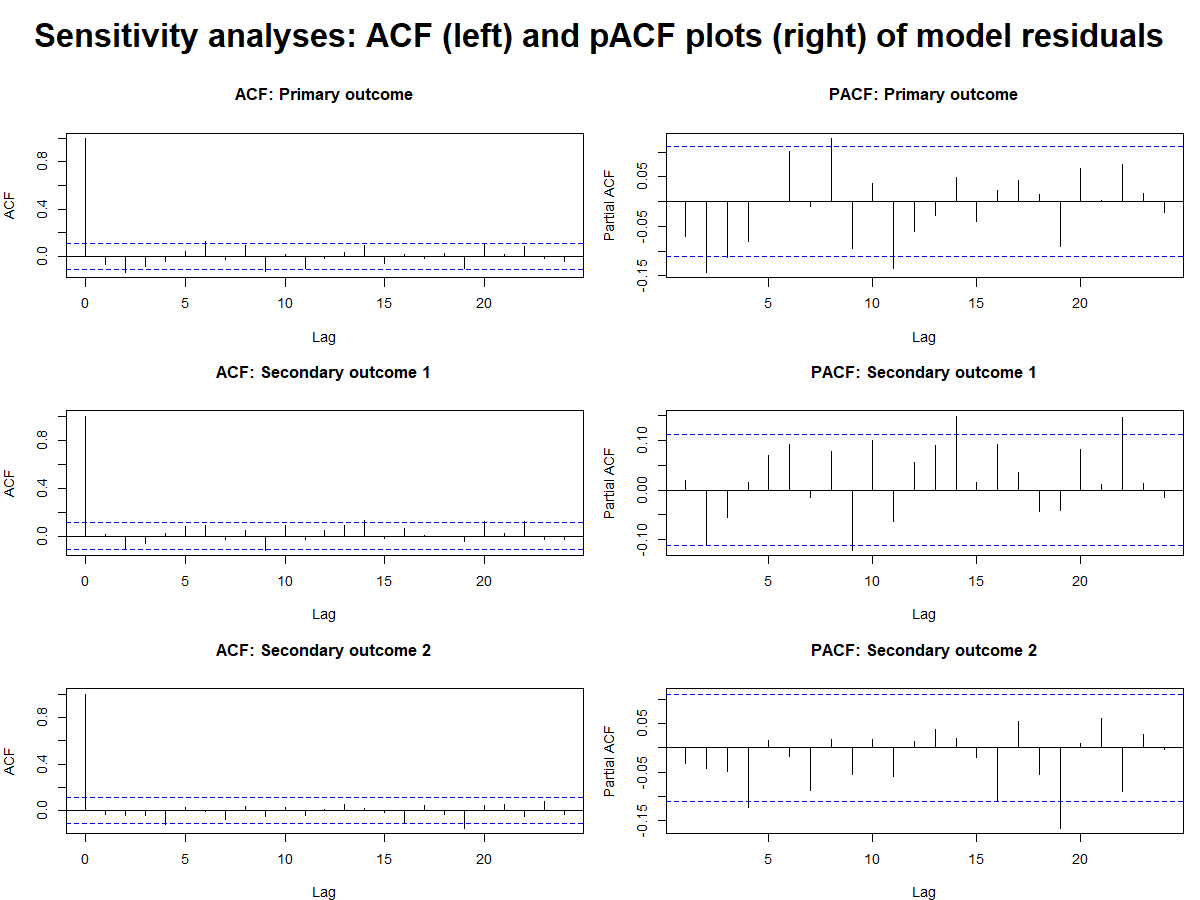


Supplementary Figure 7. Weekly number of patients in any alcohol-related treatment and by inpatient/outpatient setting (sensitivity analyses). Plotted are observed (rectangles) and predicted (lines) values from regression models. Shaded areas: red = lockdown periods (23 March 2020 to 4 May 2020; 14 December 2020 to 31 May 2021), yellow = periods with fewer restrictions (4 May 2020 to 14 December 2020; 31 May 2021 to 31 December 2021).


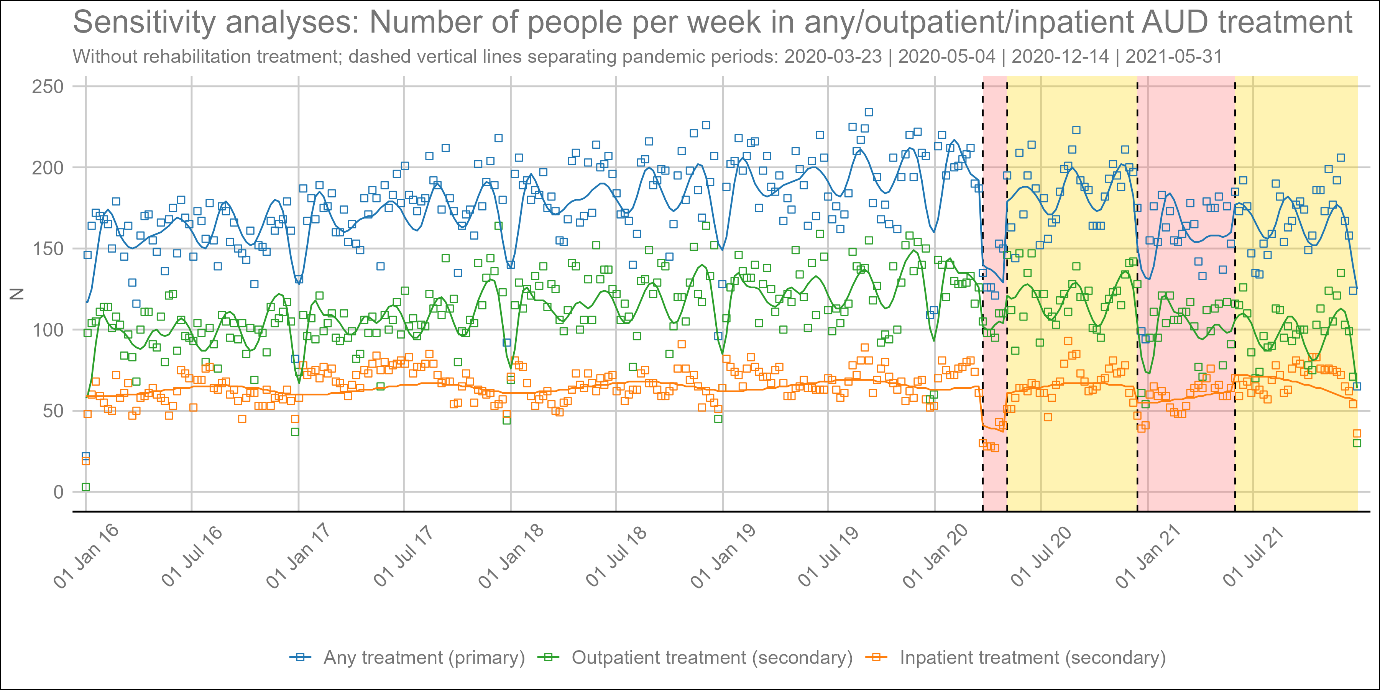


Supplementary Table 1. Definitions of time periods

| **Period** | **Dates** | **Definition** | **OxCGRT stringency index**^1^ | **Corresponding calendar week**^2^ |
| --- | --- | --- | --- | --- |
| 1 | 1 January 2016 to 22 March 2020 | Pre-covid period, including short period of few infections without severe containment measures | Before 24 Jan: 0  24 Jan to 21 Mar: 5.6 to 68.1 | 53/2015 – 12/2020 |
| 2 | 22 March to 5 May 2020 | First wave and lockdown including restrictions on travel, going out and social contact | 22 March to 5 May:  71.8 to 76.9 | 13/2020 – 18/2020 |
| 3 | 6 May to 14 December 2020 | Summer plateau with a relaxation of measures and few infections | 6 May to 14 Dec: 49.5 to 67.6 | 19/2020 – 50/2020 |
| 4 | 15 December 2020 to 30 May 2021 | Second and third wave; second lockdown including restrictions on travel, going out and social contact | 15 Dec 2020 to 30 May 2021: 75.0 to 85.2 | 51/2020 – 21/2021 |
| 5 | 31 May to 31 December 2021 | Few restrictions despite fourth and fifth waves in fall and winter | 31 May to 31 Dec 2021: 28.4 to 69.4 | 22/2021 – 52/2021 |
| ^1^ OxCGRT = Oxford COVID-19 Government Response Tracker:  Hale T, Angrist N, Goldszmidt R, Kira B, Petherick A, Phillips T, et al. A global panel database of pandemic policies (Oxford COVID-19 Government Response Tracker). Nature Human Behaviour. 2021;5(4):529-38.  Data accessed on 9 January 2025: <https://github.com/OxCGRT/covid-policy-dataset/tree/main/data>  ^2^ weeks from Monday to Sunday with the greatest overlaps with chosen dates | | | | |

Supplementary Table 2. Annual number of people utilizing AUD treatment

| **Year** | **Number of people utilizing any AUD treatment** |
| --- | --- |
| 2016 | 2,198 |
| 2017 | 2,246 |
| 2018 | 2,159 |
| 2019 | 2,180 |
| 2020 | 2,061 |
| 2021 | 1,940 |
| All years | 5,671 |

Supplementary Table 3. Model summary of sensitivity analyses

|  |  |  |  |  |  |  |  |  |  |
| --- | --- | --- | --- | --- | --- | --- | --- | --- | --- |
|  | **Primary outcome:**  **any AUD treatment^1^** | | | **Secondary outcome:**  **outpatient AUD treatment^2^** | | | **Secondary outcome:**  **inpatient AUD treatment^3^** | | |
| **Predictors** | **Estimates** | **95% CI** | **p** | **Estimates** | **95% CI** | **p** | **Estimates** | **95% CI** | **p** |
| (Intercept) | **154.46** | **147.05 – 161.87** | **<0.001** | **93.79** | **89.43 – 98.16** | **<0.001** | **62.16** | **55.20 – 69.13** | **<0.001** |
| Time | **0.20** | **0.15 – 0.26** | **<0.001** | **0.17** | **0.13 – 0.20** | **<0.001** | **0.02** | **-0.03 – 0.07** | **0.392** |
| Lockdown1 | **-49.77** | **-74.39 – -25.16** | **<0.001** | **-19.60** | **-35.47 – -3.73** | **0.016** | **-22.88** | **-33.32 – -12.45** | **<0.001** |
| TimeSinceLockdown1 | **-3.84** | **-7.48 – -0.20** | **0.039** | -1.85 | -4.00 – 0.30 | 0.092 | -1.12 | -4.11 – 1.87 | 0.461 |
| TimeSinceBetweenLockdowns | 3.90 | -0.63 – 8.43 | 0.091 | 1.50 | -1.19 – 4.18 | 0.273 | 1.32 | -2.30 – 4.95 | 0.473 |
| Lockdown2 | -14.49 | -32.86 – 3.87 | 0.122 | -3.87 | -15.25 – 7.51 | 0.504 | **-9.64** | **-19.14 – -0.14** | **0.047** |
| TimeSinceLockdown2 | -0.98 | -2.88 – 0.91 | 0.308 | -0.32 | -1.44 – 0.81 | 0.579 | -0.06 | -1.58 – 1.45 | 0.937 |
| TimeSinceAfterLockdowns | 0.39 | -1.58 – 2.35 | 0.699 | 0.13 | -1.04 – 1.30 | 0.826 | -0.55 | -2.17 – 1.07 | 0.505 |
| Smooth term (week) |  |  | **<0.001** |  |  | **<0.001** |  |  | 0.071 |
| Observations (weeks) | 314 |  |  | 314 |  |  | 314 |  |  |
| R^2^ | 0.491 |  | 0.531 |  |  |  | 0.252 |  |  |
| Note. Statistically significant effects are highlighted in bold font.  ^1^ Results of a generalized additive mixed model (GAMM) controlling for autoregression with an AR(2) term.  ^2^ Results of a generalized additive model (GAM).  ^3^ Results of a generalized additive mixed model (GAMM) controlling for autoregression with an AR(1) and MA(2) term. | | | | | | | | | |
